# Pre-resection Embolization of a Focal Lumbar Chordoma

**DOI:** 10.7759/cureus.38406

**Published:** 2023-05-01

**Authors:** Wahab A Gbadamosi, Marc Knezevic-Maragh, Priotush Talukder, Weber Smith, Edward Z Sanchez

**Affiliations:** 1 Diagnostic Radiology, Medical Center of Trinity, Trinity, USA; 2 College of Medicine, American University of Antigua, St. John's, ATG; 3 Pathology and Laboratory Medicine, Medical Center of Trinity, Trinity, USA; 4 Radiology, Northside Hospital, Saint Petersburg, USA

**Keywords:** malignant tumor resection, preoperative embolization, chronic lower back pain, chordoma, vertebral body tumor

## Abstract

Chordoma is a slow-growing local invasive tumor with high mortality and recurrence rates after surgical resection. It can affect the clivus and sacrum and rarely involve the lumbar vertebra. There is limited literature research describing lumbar embolization before surgical resection in lumbar chordoma. Thus, this case report describes an atypical patient with chronic lower back pain who presented to the hospital for worsening pain. Radiological images show an aggressive focal lesion at the second lumbar spine extending into the posterior element. The patient underwent lumbar artery embolization before surgical resection. The final pathology diagnosis confirmed a conventional chordoma. Therefore, patients with radiological imaging features of conventional chordoma may benefit from embolization prior to surgical resection to decrease intraoperative bleeding.

## Introduction

Chronic lower back pain is one of the largest epidemics in the United States, with substantial morbidity and disability [1]. Among the medical communities, differential diagnoses are considered for lower back pain, such as a ligamentous sprain, muscle etiology, degenerative disease, neuropathic, or neoplastic process. Identifying any one of these diagnoses can be challenging without medical imaging. Therefore, a patient with a primary neoplastic process can be misdiagnosed, leading to a worsened disease process if the patient does not show the classic sign and symptoms of malignancy.

Lumbar chordoma is a rare type of locally aggressive bone neoplasm that slowly grows and presents with chronic debilitating back pain [2]. Chordoma accounts for three percent of all primary bone tumors and has a high mortality rate [3]. The mainstay of treatment is the surgical resection approach. However, even after this, there are still high recurrent rates. Therefore, other adjunctive therapies may be considered. One of these options can include embolization before surgical resection. Unfortunately, there is currently limited literature on the pre-resection embolization of lumbar vertebral chordoma. Therefore, this case report aims to demonstrate an atypical patient with chronic back pain diagnosed with lumbar chordoma and discuss pre-resection embolization of the tumor.

## Case presentation

We present a case of a 70-year-old female with a history of chronic back pain that has progressively worsened over several months. The patient was in her usual state of health until approximately six months before presenting to our hospital for severe worsening lower back pain. The patient reported prior visits to multiple emergency rooms and urgent care clinics and received pain management treatment for chronic back pain. However, given the severity of the pain and inability to ambulate, the patient called emergency service and was brought to our hospital for constant and severe worsening lower back aching pain radiating to bilateral anterior thigh regions. The symptom was aggravated with ambulation with no relieving factors. The patient denies recent trauma, fever, upper respiratory illness, urinary urgency, frequency, dysuria, change in bowel habits, or unintentional weight loss. The medical history was significant for unspecified chronic back pain, coronary artery disease, hypertension, type 2 diabetes mellitus, gastroesophageal reflux, and hyperlipidemia. Surgical history was noncontributory. The patient had no known drug allergies. The home medication includes hydrocodone-acetaminophen, clopidogrel, aspirin, metoprolol, losartan, metformin, insulin, pantoprazole, and atorvastatin. Social history was significant for 38 pack years of smoking and quit approximately 20 years ago. The patient does not use alcohol or recreational drug. Family history was significant for diabetes mellitus and cardiovascular disease.

On physical examination, the temperature was 97.5F; heart rate 99 beats per minute; blood pressure 167/79 mmHg; respiratory rate 18 beats per minute; and oxygen saturation was 95% on room air. The patient appears chronically ill-appearing and obese. She was alert and oriented to self, time, place, and situation. The pupils were anicteric, round, and reactive to light. The mucosal membranes were moist and had a full range of neck motion. The lungs were clear to auscultation bilaterally. The heart sounds were normal, without gallops, murmurs, or pericardial rub. The abdomen had normal bowel sound, soft, non-distended, and no guarding or costovertebral region tenderness. The lower back demonstrates normal inspection, painful range of motion, and midline tenderness. The neurology exam shows intact cranial nerve 2-12, normal speech, bilateral lower weakness, and positive Hoffman sign bilaterally.

**Figure 1:**
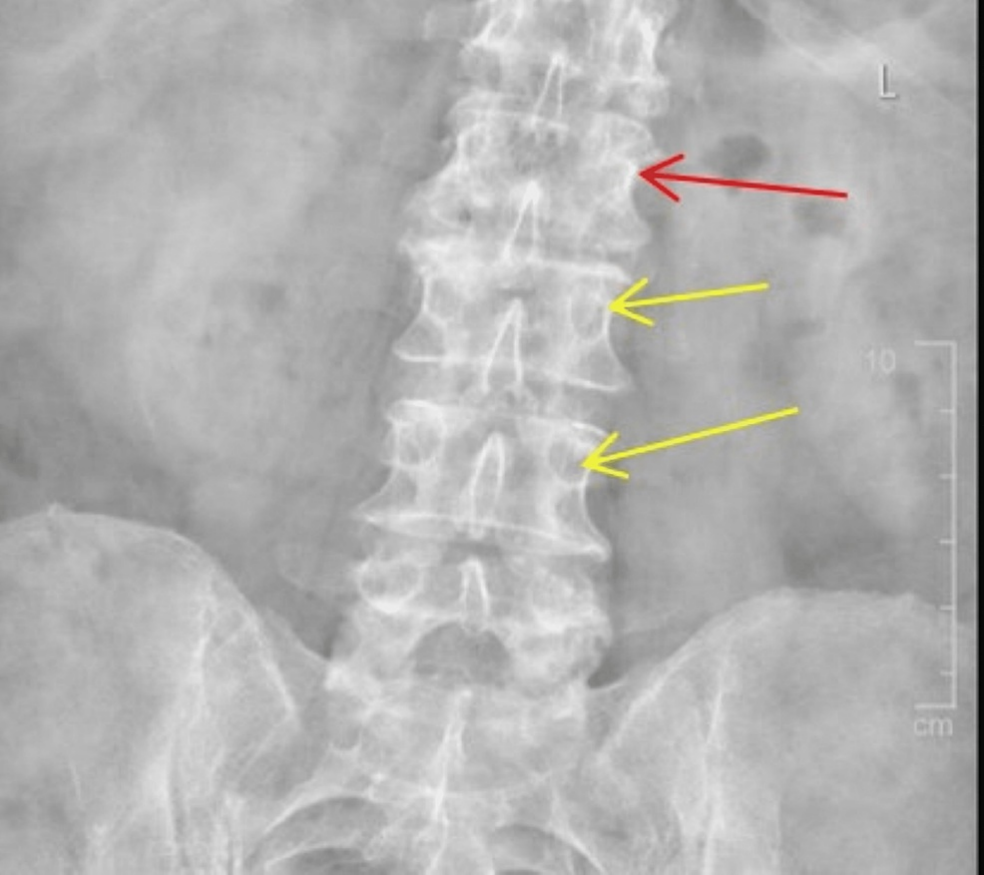
Frontal radiograph demonstrates an irregularity at the left L2 vertebral body pedicle with a wide zone of transition (red arrow) compared to the normal pedicle (yellow arrow).

**Figure 2:**
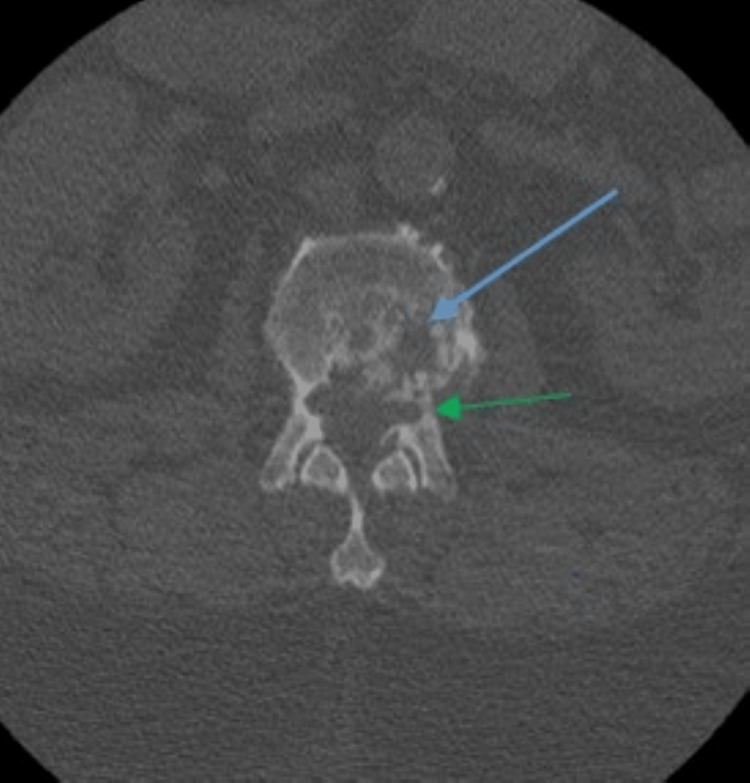
Axial computed tomography image shows a heterogeneous lytic bone erosion lesion involving the posterior left L2 vertebral body (blue arrow) with extension to the left posterior elements (green arrow).

**Figure 3:**
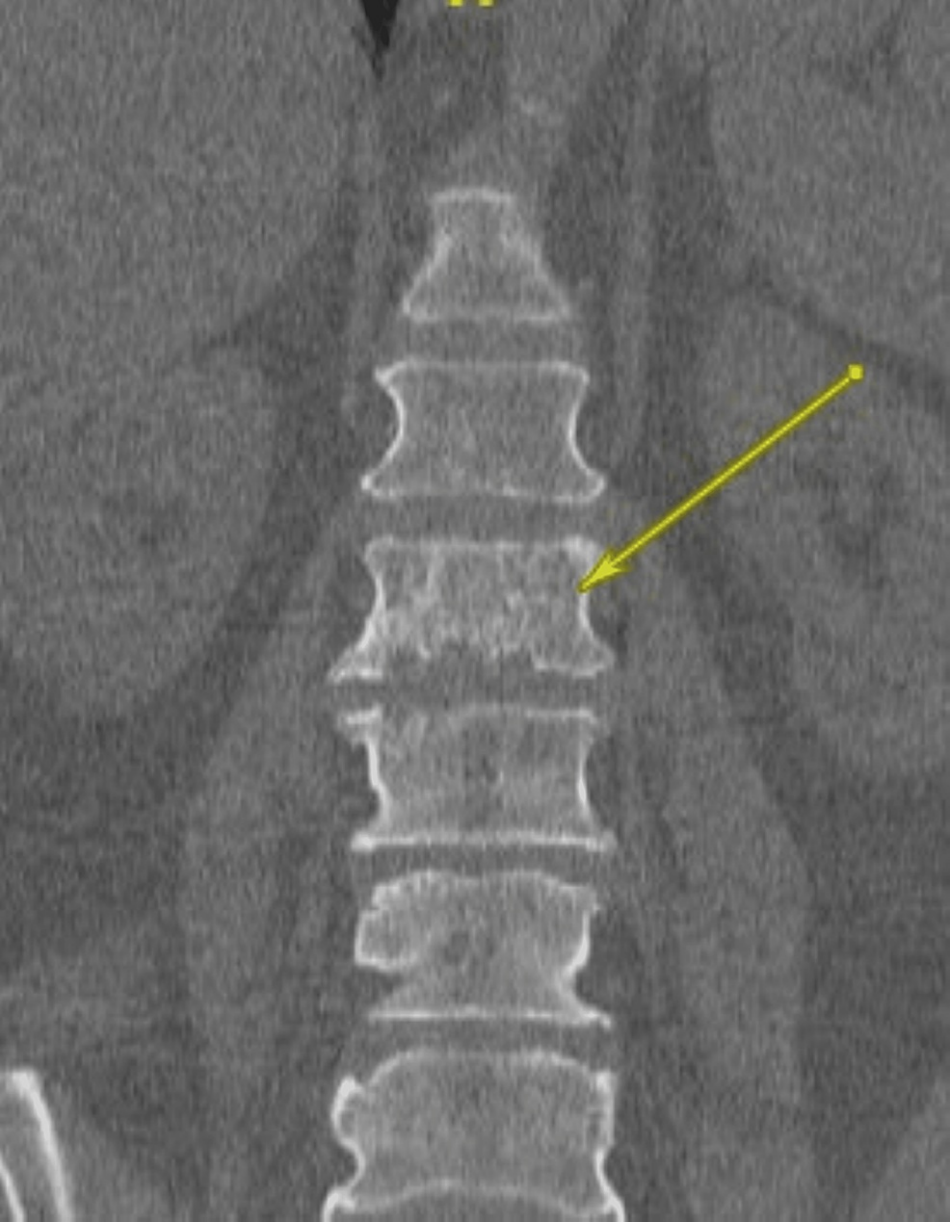
Coronal lumbar spine shows an isolated lytic erosive lesion extending into the inferior end plate (yellow arrow).

The complete blood counts demonstrate leukocytosis of 13.3uL with a neutrophil shift of 85.4% (34-71%), hemoglobin of 14.4g/dL, hematocrit 43.5%, and platelet of 186 uL. Erythrocyte sedimentation rate (ESR) 36 (0-30mm/hr), C- reactive protein (CRP) 1mg/dL (<10mg/dL), and carcinoembryonic antigen 2.4 (0-5ng/mL). The comprehensive metabolic panel was unremarkable except for 250mg/dL of elevated blood glucose. Urinalysis was significant for 1+ proteinuria with no hematuria. The initial radiology imaging of the lumbar radiograph demonstrates a second lumbar vertebral body (L2) pedicle lytic lesion (Figure 1). In addition, computed tomography (CT) of the lumbar vertebra shows an isolated heterogeneous lytic osseous lesion at the posterior left L2 vertebral body and extending into the posterior element (Figure 2 and Figure 3).

Day 1-3

**Figure 4:**
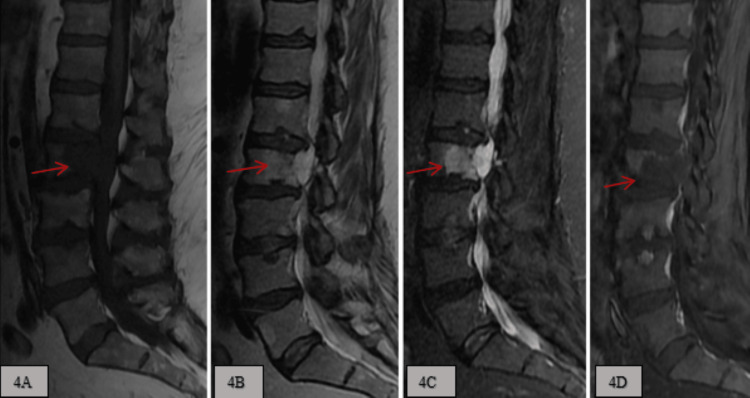
A: T1 weighted imaging, B: T2 weighted imaging, C: Short tau inversion recovery (STIR), D: T1 post-contrast imaging. These MRI sagittal image sequences demonstrate a 3.7 cm x 1.9 cm x 3.1 cm heterogenous expansile lesion at L2 vertebral body and pedicle, which is T1 hypointense, T2/STIR hyperintense, and subtle post-contrast enhancement. MRI: magnetic resonance imaging

**Figure 5:**
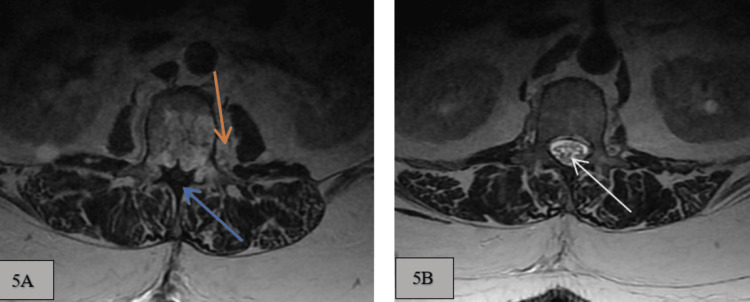
A and B: T2 weighted axial image at L1 and L2 levels. A: L2 vertebral body shows an exophytic aspect of the lesion projects into the left L2 paraspinal tissues (orange arrow) and associated anterior epidural mass effect on the central spinal canal, causing spinal canal stenosis (blue arrow). B: Normal central canal, central cord, nerve roots, and adjacent paraspinal structures at L1 vertebral level (white arrow).

The differential diagnosis was suspicious for diskitis, osteomyelitis, primary neoplasm, or metastasis. The caring primary team then consulted neurosurgery, interventional radiology, and infectious disease. The initial plan was to perform an image-guided vertebral body biopsy for diagnostic purposes with microbiology analysis. However, the follow-up trends for the ESR, CRP, and blood culture were all negative and did not follow the laboratory trend for osteomyelitis or diskitis. In addition, multiple imaging modalities, including CT chest, abdomen, and pelvis with and without contrast, showed no evidence of primary tumor or metastases in other organs. Therefore, magnetic resonance imaging (MRI) was obtained for better morphology characteristics (Figures 4A-4D and Figures 5A-5B).

Day 4-7

The MRI image findings excluded an infectious etiology and narrowed it down to a primary neoplastic process. The surgical team then consulted with interventional radiology for selective lumbar artery catheter angiogram and embolization for surgical optimization before resection.

Day 8-19

Interventional Radiology

**Figure 6:**
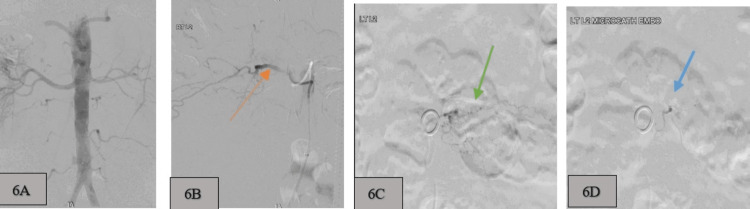
Digital subtraction angiography (DSA) images. A: Aortogram of the abdominal aorta demonstrate side branching vessels; B: Super-selective right second lumbar segmental arteriogram shows normal caliber with contrast flow (orange arrow); C: Super-selective left second lumbar segmental arteriogram shows vascular irregularity (green arrow) pre-embolization; D: Post-embolization (blue arrow) demonstrates a reduction of blood flow into the vascular irregularity previously seen in image C.

The patient underwent a diagnostic spinal angiogram and embolization. Under fluoroscopic guidance and with roadmap assistance, a super-selective microcatheter angiogram was performed. An arteriogram of the right L2 segmental artery shows good antegrade flow within the segmental arteries without tumor vascularity (Figure 6B). However, a minimal vascular irregularity was seen at the left L2 segmental artery level supplying the vertebral body tumor (Figure 6C). Therefore, the left L2 segmental artery was embolized using 500 microns embospheres (Figure 6D), followed by a coil deployment at the proximal left L2 segmental artery to decrease flow to the vertebral body tumor. Post-embolization of the super selective L2 angiogram revealed slow and stagnant flow (Figure 6D).

Neurosurgery

The neurosurgical team took the patient for an L2 lumbar vertebral body tumor resection, pediculectomy, and corpectomy a few days post-embolization. The patient tolerated the procedure well, with minimal intraoperative bleeding and no neurological compromise post-resection.

Pathology

**Figure 7:**
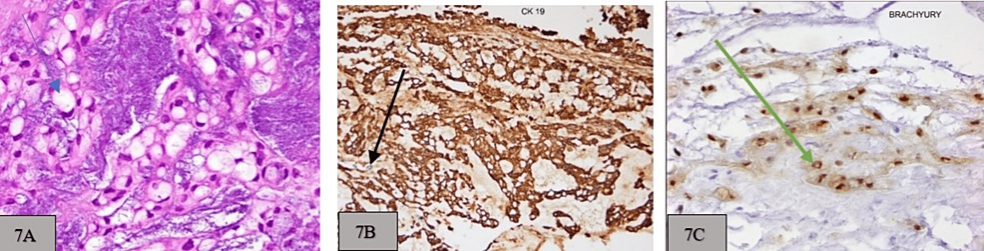
A: Hematoxylin and eosin stains demonstrate clear bubbly cytoplasm with surrounding trabeculae nests and a sheet of tumor cells within a myxoid matrix (blue arrow); B: Intracellular positive cytokeratin 19 (black arrow); C: Specific nuclear brachyury expression (green arrow).

Multiple pathological stains were performed, which included hematoxylin, eosin, cytokeratin, and brachyury. The resected fragments of bone were composed of large epithelioid cells arranged in nests and cords with an extensive extracellular myxoid matrix, small hyperchromatic nuclei, and abundant vacuolated cytoplasm (Figure 7A). Immunohistochemical stains demonstrate the tumor cells were positive for brachyury, calcium-binding cytosolic protein (S100), cytokeratin 19 (CK19), and cytokeratin cam 5.2 protein (Figures 7B-7C). These pathological findings were consistent and a hallmark of conventional chordoma.

Oncology

The patient was scheduled for an outpatient oncology follow-up, further imaging workup with PET/CT, and a discussion with radiation oncology.

Day 20

She was discharged to an acute rehabilitation facility for recovery.

## Discussion

Vertebral chordoma is a rare type of bony malignant neoplasm that is slow-growing and locally aggressive, with an overall five-year survival of approximately fifty percent [2]. The incidence of chordoma is estimated to be 1 per 1,000,000 people, and about 300 new cases occur in the United States each year [3]. Chordoma primarily affects patients between the ages of 40-75 years and is seen predominantly in males compared to females in a ratio of 2:1 [2,3]. During 1800-1900, the medical community generated multiple hypotheses regarding the origin of the lesion [4,5]. In 1846 German pathologist Rudolf Virchow noted a small slimy growth at the dorsum sella on autopsy and called it chordomata [4,5]. Additionally, Dr. Micotti used an exploratory puncture to diagnose sacrococcygeal chordoma in 1922 [5]. After numerous medical research during 1900, the medical community concluded that chordoma originated from notochord remnant tissue and was an aggressive malignant tumor [4,5].

Chordoma accounts for twenty percent of primary spinal tumors and only three percent of all bone neoplasms [2,3]. Chordoma can occur anywhere along the vertebral column. Thirty percent occurred in the skull base clivus, twenty percent at the cervical, thoracic, or lumbar vertebral body, and fifty percent in the sacrum [4]. World health organization (WHO) grouped soft tissue and bone tumor chordoma into three classifications based on histological appearance and characteristics. These are conventional, dedifferentiated, and poorly differentiated. The conventional type is the most common and appears as bubbly cystic cell types with an expression of brachyury protein as a hallmark [3,6]. The second is called the dedifferentiated type. It is scarce and composed of heterogeneous cell types which may or may not express brachyury protein [3,7]. Finally, the poorly differentiated type has a deletion of a gene called SWI/SNF-related matrix-associated actin-dependent regulator of chromatin subfamily B member 1 (SMARCb1 or INI1), which codes for SWI/SNF protein complex to help with chromatin remodeling [3,7-10]. The dedifferentiated and poorly differentiated both have the worst prognosis [9,10]. Our patient's tumor cell type was consistent with the conventional type.

The patient clinical presentation depends on the anatomical site involved. Due to the slow-growing features of chordoma, symptoms tend to be observed when there has been a significant mass effect or invasion of the surrounding adjacent structures. The differential diagnosis for chordoma includes chondrosarcoma, plasmacytoma, ecchordosis physaliphora, benign notochord cell tumor (BNCT), and atypical notochordal cell tumor [7,11]. These differential diagnoses are separated based on pathological tissue samples [7]. Furthermore, medical imaging can help delineate the etiology of chronic back pain and help identify a primary bone lesion. CT and MRI are the core imaging modalities used for imaging workup for chordoma. CT is used to assess the degree of bone involvement, and MRI provides better resolution of adjacent structures and the tumor signal for preoperative diagnosis [11]. On CT, chordoma is a destructive irregular expansile lytic lesion with a soft tissue component. MRI shows a low to intermediate signal on T1, a high signal on T2, and heterogenous to minimal enhancement on T1 post gadolinium contrast [11]. Finally, pathology is the definitive method of diagnosing chordoma. It appears as a lobulated intraosseous mass with different histopathology characteristics depending on the subset as lobules containing a short nest of large epithelioid cells with clear to light eosinophilic, vacuolized cytoplasm, abundant myxoid matrix, and increased mitosis [7]. Therefore, the radiological and pathological features complement each other and help establish the diagnosis of the chordoma subtype. In our patient, the radiological and pathological features matched with the conventional type of chordoma, which is the most common type but not at a common vertebral location.

Multiple ongoing clinical trials assess the safety and efficacy of numerous drug-directed therapies in cases of advanced and metastatic chordoma [12]. However, the current mainstay of chordoma treatment is surgical resection when feasible with radiotherapy [11,12]. Moreover, a total resection can be impossible given the vertebral location, leading to a high recurrence rate. The prognosis is typically poor due to this tumor's significantly locally aggressive nature, with an overall 10-year survival of approximately forty percent [11]. Therefore, in addition to these treatment regimens, a pre-resection embolization may be considered in appropriate patients. Chordoma mostly does not have significant tumoral vascularity, but if a vascularity lesion is suspected on CT or MRI, a pre-surgical resection angiography and embolization can be suggested [13,14]. These embolization techniques are generally used to reduce intraoperative blood loss and make tumor margins more easily identifiable. Unfortunately, there is limited to no robust clinical research on surgical pre-resection embolization for lumbar chordoma. However, within this limitation, Yang et al. demonstrated a retrospective study of 30 patients with sacral chordoma who underwent posterior approach resection after transcatheter arterial embolization, which significantly decreased intraoperative blood loss and facilitated maximal removal of the lesion when surgery was performed within 24-48 hours post embolization [14]. Therefore, a pre-resection embolization can be a valuable adjunctive treatment option that may be considered for these patients when clinically warranted. Our patient underwent a diagnostic angiogram with embolization of the left L2 segmental artery with embosphere before surgical excision with no significant intraoperative blood loss, good tumor margin resection, and no neuraxial compromise.

## Conclusions

Lumbar chordoma is a rare, slow-growing, aggressive malignant tumor with local destructive features leading to chronic back pain. The epidemic of chronic back pain in adult patients and the long wait time to see a specialist can lead to misdiagnoses and bad surgical outcomes due to significant neoplasm invasion at the time of diagnosis. The primary treatment is surgical resection; however, currently, there is limited to no well-established literature on pre-resection embolization to show benefits or adjunctive therapy to these patients. Therefore only future research can identify patients with medical imaging suggestive of lumbar chordoma and correlate how they perform post-embolization and throughout the perioperative stages, which might help improve prognosis.
